# Regulatory Role of Hexokinase 2 in Modulating Head and Neck Tumorigenesis

**DOI:** 10.3389/fonc.2020.00176

**Published:** 2020-03-03

**Authors:** Wan-Chun Li, Chien-Hsiang Huang, Yi-Ta Hsieh, Tsai-Ying Chen, Li-Hao Cheng, Chang-Yi Chen, Chung-Ji Liu, Hsin-Ming Chen, Chien-Ling Huang, Jeng-Fan Lo, Kuo-Wei Chang

**Affiliations:** ^1^Institute of Oral Biology, School of Dentistry, National Yang-Ming University, Taipei, Taiwan; ^2^Department of Dentistry, School of Dentistry, National Yang-Ming University, Taipei, Taiwan; ^3^Cancer Progression Research Center, National Yang-Ming University, Taipei, Taiwan; ^4^Department of Oral and Maxillofacial Surgery, MacKay Memorial Hospital, Taipei, Taiwan; ^5^Department of Medical Research, MacKay Memorial Hospital, Taipei, Taiwan; ^6^School of Dentistry and Department of Dentistry, National Taiwan University Medical College and National Taiwan University Hospital, Taipei, Taiwan; ^7^Department of Health Technology and Informatics (HTI), The Hong Kong Polytechnic University (PolyU), Kowloon, Hong Kong; ^8^Department of Stomatology, Taipei Veterans General Hospital, Taipei, Taiwan

**Keywords:** head and neck cancer, hexokinase 2, cellular malignancy, metabolic shift, therapeutic efficacy

## Abstract

To support great demand of cell growth, cancer cells preferentially obtain energy and biomacromolecules by glycolysis over mitochondrial oxidative phosphorylation (OxPhos). Among all glycolytic enzymes, hexokinase (HK), a rate-limiting enzyme at the first step of glycolysis to catalyze cellular glucose into glucose-6-phosphate, is herein emphasized. Four HK isoforms, HK1-HK4, were discovered in nature. It was shown that HK2 expression is enriched in many tumor cells and correlated with poorer survival rates in most neoplastic cells. HK2-mediated regulations for cell malignancy and mechanistic cues in regulating head and neck tumorigenesis, however, are not fully elucidated. Cellular malignancy index, such as cell growth, cellular motility, and treatment sensitivity, and molecular alterations were determined in HK2-deficient head and neck squamous cell carcinoma (HNSCC) cells. By using various cancer databases, HK2, but not HK1, positively correlates with HNSCC progression in a stage-dependent manner. A high HK2 expression was detected in head and neck cancerous tissues compared with their normal counterparts, both in mouse and human subjects. Loss of HK2 in HNSCC cells resulted in reduced cell (*in vitro*) and tumor (*in vivo*) growth, as well as decreased epithelial-mesenchymal transition–mediated cell movement; in contrast, HK2-deficient HNSCC cells exhibited greater sensitivity to chemotherapeutic drugs cisplatin and 5-fluorouracil but are more resistant to photodynamic therapy, indicating that HK2 expression could selectively define treatment sensitivity in HNSCC cells. At the molecular level, it was found that HK2 alteration drove metabolic reprogramming toward OxPhos and modulated oncogenic Akt and mutant TP53-mediated signals in HNSCC cells. In summary, the present study showed that HK2 suppression could lessen HNSCC oncogenicity and modulate therapeutic sensitivity, thereby being an ideal therapeutic target for HNSCCs.

## Introduction

Most cancers have acquired the same set of functional capabilities during tumorigenesis including sustained proliferative signal, evaded growth suppressors, resisted cell death, sustained angiogenesis, and deregulated cellular energetics (1). In the aspect of energy production, glucose is a major source of bioenergetics and biomolecules used to maintain cellular homeostasis. Glucose molecules could be metabolized by glycolysis to generate pyruvate in cells; the pyruvate could be either oxidatively metabolized to generate up to 38 ATPs through the process of oxidative phosphorylation (OxPhos) or be reductively converted into organic acids or alcohols by fermentation producing only 2 ATP (2). In general, normal cells in non-malignant tissues developed an evolutionary choice of “Pasteur effect” to allow a system to fine tune cell metabolism in unfavorable environment (3, 4). On the contrary, cancer cells exhibited a unique metabolic system by taking advantage of aerobic glycolysis for demands of energy and biomolecules regardless of oxygen status; in this way, cancer cells preferentially drive their metabolism away from OxPhos toward glycolysis, so-called Warburg effect (5).

Previous studies have sought to target different cancer-specific metabolic pathways, in particular glycolysis, for development of antitumor treatments (6–9). There are three irreversible reactions in glycolysis. It is well-known that the glycolytic rate is limited by the phosphorylation state of glycolytic enzymes hexokinases (HKs), because at the first step of glycolysis, HKs catalyze the intake glucose into glucose-6-phosphate (G-6-P) (10). Hexokinase–mediated catalysis is ATP dependent and a key determinant for the direction and magnitude of glucose flux within cells; thus, it would more efficient to target glucose metabolism in cancer cells by cutting down this earliest step of glucose flux (11). Four HK isoforms including HK1, HK2, HK3, and HK4 encoded by different genes were found in nature. Among them, HK1 is ubiquitously expressed in most mammalian tissues, and HK2 is detected in adipose, skeletal, and cardiac muscles, whereas HK3 and HK4 show relatively low expression in mammalian tissues (12). In the aspect of protein structure, two catalytic domains, both in N- and C-terminal portions, are detected in HK2, resulting in greater glycolytic rate compared with HK1-mediated glycolysis, whereas only one catalytic domain was found at the carboxyl terminal part in HK1 protein (13). In clinic, HK2 is rarely expressed in normal tissues as a high level of HK2 expression was detected in many solid tumors (14) and is associated with elevated progression, poorer overall survival, and treatment resistance in breast cancer (both primary and metastatic kinds) (15–18), bladder cancer (19), cervical squamous cell carcinoma (20), colorectal cancer (21), epithelial ovarian tumors (22), glioblastoma multiforme (23), hepatocellular carcinoma (24), laryngeal squamous cell carcinoma (25), lung cancer (26), neuroblastoma (27), neuroendocrine tumor (28), pancreatic cancer (29), and prostate cancer (30), making it an excellent target for development of anticancer therapy. Moreover, given its selective expression in tumors, HK2 has drawn increasing attention on its clinical implications forming the basis of fluorodeoxyglucose F-18 positron emission tomography imaging, a widely used clinical procedure to detect tumors. Indeed, a recent meta-analysis provides evidence that HK2 could be a marker to predict the risk of all-cause mortality and cancer progression in patients with tumors of the digestive system (31).

Numbers of molecular cues including signal transducer and activator of transcription 3 (32–35), protein kinase B (PKB), also known as Akt (19, 36–38), mammalian target of rapamycin (38–40), and PH domain leucine-rich repeat protein phosphatase (41), as well as autophagy (42, 43), have been found to control HK2-mediated metabolic alterations, thereby regulating tumor identity. Moreover, HK2 contains an N-terminal hydrophobic domain allowing it to bind to outer mitochondrial membrane voltage-dependent anion channel (VDAC) protein, thereby resulting in Bax-mediated apoptosis. HK2–VDAC interaction could also provide kinetic benefits in HK2 exhibiting greater affinity to ATP over G-6-P, suggesting that HK2 could regulate oncogenicity via modulation of mitochondrial physiology (44–47). More recent studies further discovered that p53-inducible protein TIGAR (Tp53-induced Glycolysis and Apoptosis Regulator), Akt, and ER stress sensor kinase could regulate mitochondrial HK2 localization (48–51). Interestingly, mitochondrial TIGAR–HK2 complex upregulated HK2 and hypoxia-inducible factor 1 activity limiting reactive oxygen species production and protecting from tumor cell death under hypoxic condition implying that p53 could be an essential key regulator for HK2-mediated oncogenesis (26, 30, 48). In addition to signaling factors, HK2 regulated by epigenetic mediators including microRNAs (32, 52–55), long non-coding RNAs (56–58), and histone/DNA methylation (59) are also important to modulate HK2-mediated tumorigenicity. Taken together, HK2 seems to be a master promoting factor in controlling carcinogenesis in different cancers; to date, however, the role of HK2 in controlling head and neck squamous cell carcinoma (HNSCC) development was rarely focused. The present study was therefore conceived to determine cellular, molecular, and physiological alterations of HNSCC cells in response to HK2 silencing.

## Materials and Methods

### Basic Reagents, Cells, and Animal and Human Protocols

All experimental reagents including 4-nitroquinoline 1-oxide (4-NQO), 3-(4,5-Dimethylthiazol-2-yl)-2,5-diphenyltetrazolium bromide (MTT), cisplatin (CDDP), and 5-fluorouracil (5-FU) were purchased from Sigma. The HNSCC cell lines, clinical human HNSCC tissues, and animal procedure were obtained and described elsewhere (60, 61).

### Establishment of HK2 Silencing HNSCC Cells

Three plasmids encoding small hairpin RNAs (shRNAs) targeting HK2 gene were purchased from the National RNAi Core Facility (NRCF), Academia Sinica, Taipei, Taiwan. The targeting sequences were as listed: 5-GCCTGGCTAACTTCATGGATA-3 (A), 5-CCGTAACATTCTCATCGATTT-3 (B), and 5-GCTTGAAGATTAGGTACTATC-3 (C) (NRCF). The control nucleotide sequence of shRNA was 5-GCGGTTGCCAAGAGGTTCCAT-3, which was the random sequence that targets luciferase (shLuc) mRNA. Lentiviral vectors containing shHK2/shLuc were generated in 293T cells.

### Cellular and Molecular Assays

Cell growth (MTT and trypan blue exclusion assays), cell cycle, annexin V–fluorescein isothiocyanate–based cell apoptosis, Transwell-based cell migration/invasion, drug resistance (IC50 determination), quantitative real-time polymerase chain reaction (qRT-PCR), Western blot, aldehyde dehydrogenase (ALDH) activity, immunostaining analysis, human phospho-antibody array, and XF Metabolic Assay analyses were performed as previously described at Instrumentation Resource Center, National Yang Ming University (61, 62). Primers for qRT-PCR analysis (Supplementary Table 1) and antibodies used for protein assays (Supplementary Table 2) are listed. ImageJ (National Institute of Health, Bethesda, Maryland, United States) was used for quantification.

### Aminolevulinic Acid–Photodynamic Therapy Treatment

5-Aminolevulinic acid (ALA) 1 mM was made by diluting 1 M ALA stock with serum-free medium and neutralized to pH 7.2 with NaOH immediately before use. The total number of 5 × 10^5^ cells were incubated with 1 mM ALA for 3 h, and the medium was replaced with 100 μL phosphate-buffered saline to prevent thermal injury by the light. The cells were then exposed to various doses of light source (30.33 mW/cm^2^) with a 635-nm wavelength emission of red light. After exposure, cells are replaced with culture medium and cultured for 24 h. Relative viable cells were determined by the MTT method.

### Measurement of Lactate, ATP, and PDH Activity

Extracellular lactate, intracellular ATP level, and pyruvate dehydrogenase (PDH) activity were determined previously (62). Cell number or genomic DNA content was used to normalize detections.

### Xenografic Model

The HNSCC cells 1 × 10^6^ to 4 × 10^6^ and 1 μL of each medium were mixed and subcutaneously injected into the back of nude mice. Tumor volume and size were recorded.

### Statistical Analysis

All analyses were statistically determined using Microsoft Excel 2013 (Microsoft, Redmond, WA, United States)/Prism 5 (GraphPad, San Diego, CA, United States). All quantifications are presented as mean ± SEM. A significant difference was defined as *p* < 0.05.

## Results

### HK2 Expression Defines HNSCC Progression in Clinic

**Figure 1:**
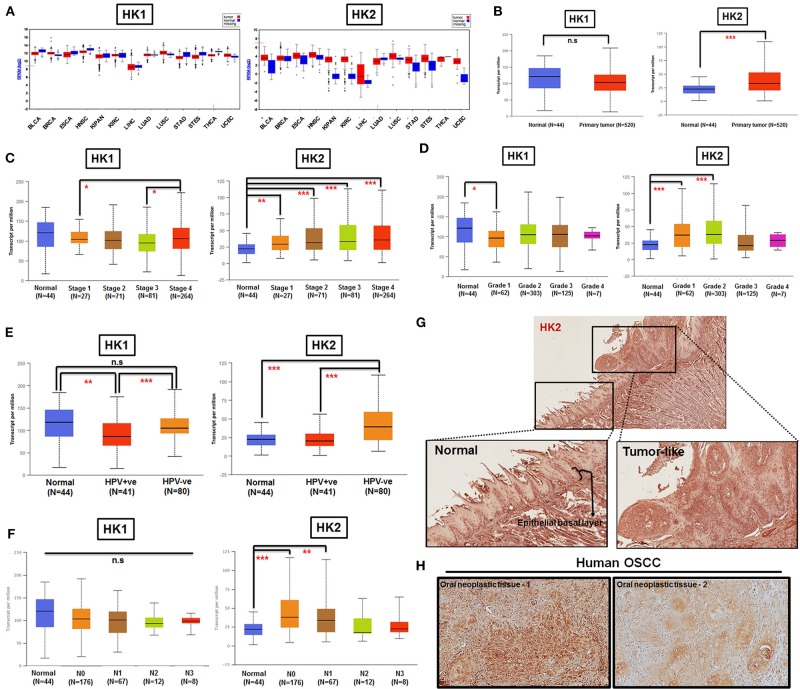
HK2 level positively correlates with HNSCC progression in clinic. **(A)** HK1, but not HK2, mRNA expression is enriched in most human tumors by using Firebrowser database. BCLA, bladder urothelial carcinoma; BRCA, breast invasive carcinoma; ESCA, esophageal carcinoma; HNSCC, head and neck squamous cell carcinoma; KIPAN, Pan-kidney cohort (KICH+KIRC+KIRP); KIRC, kidney renal clear cell carcinoma; LIHC, liver hepatocellular carcinoma; LUAD, lung adenocarcinoma; LUSC, lung squamous cell carcinoma; STAD, stomach adenocarcinoma; STES, stomach and esophageal carcinoma; THCA, Thyroid carcinoma; UCEC, uterine corpus endometrial carcinoma. **(B)** Statistical analysis for HK1 and HK2 mRNA levels in normal and primary HNSCC tissues as well as tumor tissues, classified by clinical **(C)** stages, **(D)** grade, **(E)** HPV infection status, and **(F)** nodal metastasis status from UALCAN database. Immunohistochemical analysis for HK2 in **(G)** in 12-week 4-NQO–treated mouse tongue and **(H)** human oral squamous cell carcinoma (hOSCC) tissues. Data are shown as mean ± SEM. ***p < 0.001, **p < 0.01, *p < 0.05, and n.s., non-significant.

By taking advantage of The Cancer Genomic Atlas–based UALCAN, Firebrowse, and The Human Protein Atlas (www.proteinatlas.org) databases (63–65), it was found that HK2 transcript, in comparison with HK1, is enriched in most primary tumor tissues compared with their normal counterparts (Figures 1A,B). Further analysis for HK1 and HK2 expression in HNSCCs indicated that (i) HK2 expression, but not HK1, is positively correlated with tumor stages and grades (Figures 1C,D); (ii) HK2 level, but not HK1, is upregulated in HPV^−^ HNSCC tissues (Figure 1E); and (iii) HK2 expression, but not HK1, is increased in early metastatic HNSCCs [with one to three axillary lymph nodes (N1)] (Figure 1F). Furthermore, immunohistochemical analysis showed that HK2 is strongly detected in 4-NQO–induced mouse tongue neoplastic lesions compared with normal oral epithelium (Figure 1G), as well as human oral squamous cell carcinoma (Figure 1H). Surprisingly, differential HK1 level could better stratify cancer-specific survival rates than HK2 in HNSCC patients (Supplementary Figure 1), suggesting that HK1 and HK2 might play differential roles during HNSCC tumorigenesis. Taken together, these analyses suggest that HK2 serves as a rather early prognostic indicator in the majority of HNSCC population.

### HK2 Loss Modulates HNSCC Cell Growth and Motility

**Figure 2:**
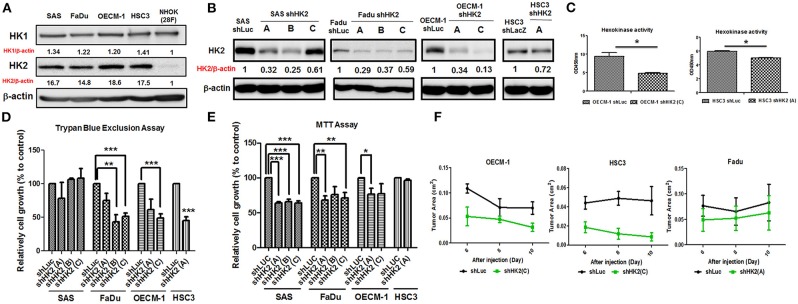
Differential HNSCC cell growth and HNSCC-bearing xenografic tumor growth in response to HK2 loss. Respective Western blot analysis for HK1 and HK2 protein expression in **(A)** HNSCC and NHOK cells and **(B)** shRNA-mediated HK2-silencing HNSCCC cells. **(C)** Colorimetric hexokinase activity assay showed functional deficient in HK2-silencing OECM-1 and HSC3 cells. Cell growth was examined in HK2-silencing HNSCC cells using **(D)** trypan exclusion and **(E)** MTT assays. **(F)** HK2 loss downregulated HNSCC-bearing xenografic tumor growth. shLuc vector is used as a control plasmid. Data are shown as mean ± SEM (*N* ≥ 3). ***p < 0.001, **p < 0.01, and *p < 0.05.

In consistent with former findings in other cancer tissues, HK2, both in mRNA and protein level, is highly expressed in tested HNSCC cell lines (Supplementary Table 3) compared to normal human oral keratinocytes (NHOKs) (Figure 2A and Supplementary Figure 2). A wide spectrum of cellular alterations and their potential underlying regulatory cues in response to HK2 loss were next focused on. As shRNA-mediated HK2-deficient HNSCC cells were successfully established without apparent morphological change (Figures 2B,C and Supplementary Figure 3), it was demonstrated that, by using both trypan blue exclusion (Figure 2D) and MTT (Figure 2E) assays, HK2-silencing resulted in decreased cell growth in HNSCC cells. Further analysis found no significant cell cycling change and cell apoptosis (data now shown) in HK2-silencing HNSCC cells compared with control cells, suggesting that HK2 could possibly control cell growth via self-protective machinery, such as autophagy (42, 43). *In vivo* growth for HK2-silencing tumors was also analyzed, and it was shown that HK2 loss resulted in significantly smaller xenografic tumors compared with control tumors (Figure 2F).

**Figure 3:**
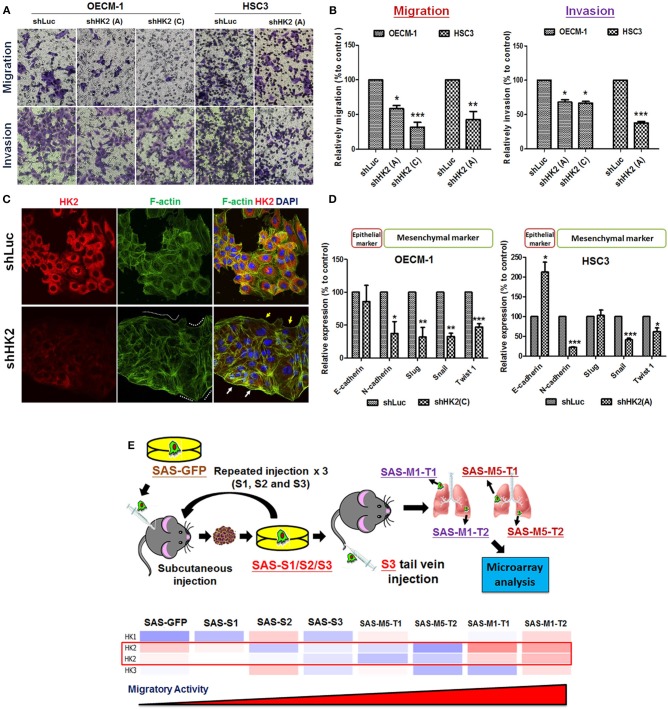
Cell motility changes in HK2-silencing HNSCC Cells. **(A,B)** Transwell cell migration and invasion assays were performed in HK2-silencing HNSCC cells. HK2 knockdown led to significantly less migration and invasion in HNSCC cells. The changes of cell motility could result from **(C)** rearrangement of lamellipodia-like (dot-lined) and filopodia-like F-actin structures. HK2-silencing OECM-1 cells showed heterogeneous population, whereas cells with higher HK2 expression exhibited more condense F-actin structure (white arrows), while cells with less HK2 expression displayed little F-actin staining; and **(D)** altered EMT-associated mRNA expression. **(E)** Microarray analysis showed an increasing HK2, but not HK1 and HK3, mRNA expression in *in situ* injected (S1/2/3) and distant metastatic (M1/M5) HNSCC derived tumors. Data are presented as mean ± SEM (*N* ≥ 3). ***p < 0.001, **p < 0.01, and *p < 0.05.

Potential impact of HK2 loss in regulating cell motility was also analyzed by using Transwell-based assays (62). The results indicated that HK2 deficiency led to significant decrease of cell migration and invasion in OECM-1 and HSC3 clones (Figures 3A,B). At cellular and molecular levels, lactate level, cytoskeletal organization and epithelial–mesenchymal transition (EMT) could modulate cell motility (66–68). While a slight, but not significant, increase in extracellular lactate production was detected in HK2-silencing HNSCC cells (Supplementary Figure 4), immunofluorescence staining analysis implied that lamellipodia-like F-actin (dot-lined in Figure 3C) was predominantly found in HK2-deficient HNSCC cells in comparison with control cells those contain more evident filopodia-like protrusions. Interestingly, as shRNA-mediated HK2 manipulation seems to heterogeneously silence HK2 protein, HNSCC cells with greater HK2 loss (white arrows in Figure 3C) showed more and compacted F-actin staining signals compared with cells with less HK2 expression (yellow arrows in Figure 3C), revealing that HK2 loss potentially reduces F-actin level in a single-cell basis. Moreover, increased epithelial marker E-cadherin accompanied with decreased mesenchymal markers N-cadherin, Snail, and Twist was detected in mRNA level (Figure 3D), further supporting the important role of EMT in HK2-mediated cell motility alteration. The role of HK2 in regulating tumor metastasis *in vivo* was further defined in a previously established primary and metastatic xenografic tumor model (60). By using microarray analysis, it was found that HK2, but not HK1 and HK3, is enriched in highly migrating cells in this model (Figure 3E). In brief, multiple molecular factors play key roles in regulating cell movement upon HK2 loss.

### HK2 Expression Determines Treatment Resistance in HNSCCs Through Regulations of Stemness and Differentiation

**Figure 4:**
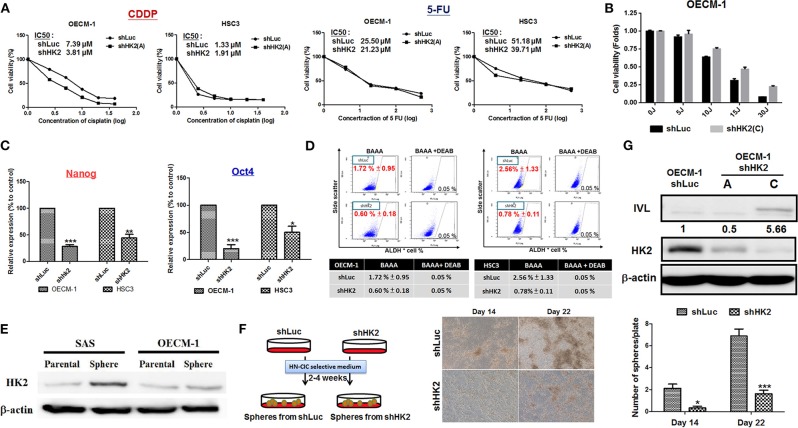
HK2 loss modulated therapeutic sensitivity in HNSCC Cells. **(A)** Decreased IC50 for clinical chemotherapeutic agent CDDP and 5-FU, whereas **(B)** greater resistance in response to photodynamic therapy was detected in HK2-deficient HNSCC cells. This effect is probably regulated via decreased **(C)** Nanog/Oct4 mRNA expression and/or **(D)** ALDH activity. While **(E)** HK2 is highly expressed in SAS and OECM-1 derived sphere cells compared with parental cells, **(F)** HK2 loss resulted in decease of sphere formation. **(G)** Upregulation for cellular differentiation marker involucrin (IVL) protein was detected by Western blot analysis in HK2-silencing HNSCC cells. Data are presented as mean ± SEM (*N* ≥ 3). ***p < 0.001, **p < 0.01, and *p < 0.05.

The sensitivity for chemotherapeutic drugs, CDDP and 5-FU, as well as for photodynamic therapy (PDT), in HK2-silencing HNSCC cells was next examined. The results showed a common pattern of decreased half maximal inhibitory concentration (IC50) level for CDDP and 5-FU in HK2-deficient HNSCC cells compared with control cells (Figure 4A). Photodynamic therapy is an approved adjuvant treatment to lessen the disease mass of various premalignant and malignant lesions, including HNSCCs (69, 70). Photodynamic therapy–mediated cell apoptosis could be induced under numbers of physiological and pathological conditions, and one of the main advantages of PDT treatment is the higher selectivity to target transformed cells over normal cells (71). As HK2 loss could potentially reduce cell malignancy in HNSCC cells, it is expected that HK2-silencing HNSCC cells are less sensitive to PDT treatment (Figure 4B). At molecular level, previous study has shown that HK2 is essential for tumor initiation and survival in Kras-driven mouse lung cancer and ErbB2-driven mouse breast cancer, indicating that HK2 could be important to regulate cancer stemness (24). Moreover, it was also shown that elevated stemness could be a key factor underlying chemoresistance in HNSCCs (72). Consistent with this notion, stemness indicators Nanog and Oct4 mRNA (Figure 4C) and ALDH activity (Figure 4D) were downregulated in HK2-silencing HNSCC cells. Further analysis showed that HK2 protein is enriched in stem-like/high-grade sphere cells (Figure 4E), and strikingly, HK2 loss resulted in significantly less sphere number in selective medium used to culture head and neck cancer–initiating cells (Figure 4F). On the other hand, epithelial differentiation marker involucrin in OECM-1 cells with greater HK2 loss (clone C) was highly detected (Figure 4G), further supporting that HK2 change could regulate stemness and differentiation status, which modulate treatment sensitivity in HNSCC cells.

### Molecular Alterations and Metabolic Shift in HK2-Silencing HNSCC Cells

**Figure 5:**
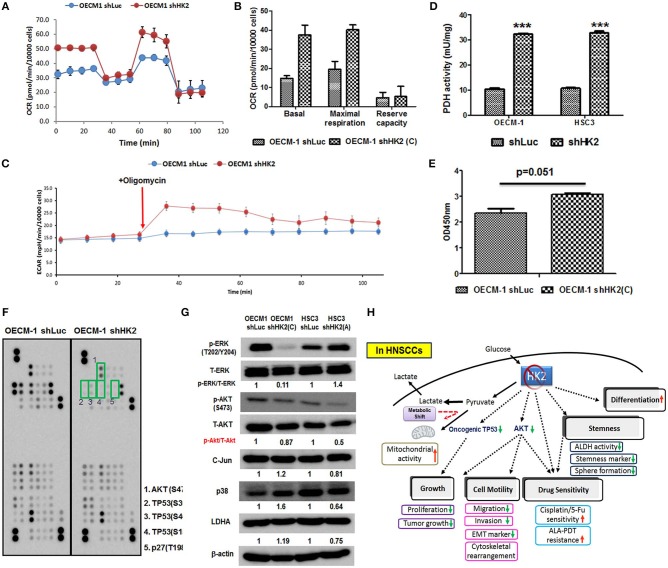
Metabolic and molecular changes in HK2-silencing HNSCC cells. **(A,B)** Seahorse metabolic analysis showed an elevated basal respiration and maximal respiration in HK2 deficient OECM-1 cells. **(C)** Sharp increase of ECAR upon addition of ATPase inhibitor oligomycin and **(D)** increased pyruvate dehydrogenase activity reflected that HK2 loss triggers HNSCC cells toward mitochondrial metabolism. **(E)** ATP level was slightly increased in HK2-silencing OECM-1 cells. **(F)** Human protein array analysis for phosphorylated proteins differentially expressed in HK2-silencing OECM-1 cells was performed. Green boxes indicated oncogenic phosphorylated molecules: (1) Akt (S473), (2) TP53 (S392), (3) TP53 (S46), (4) TP53 (S15), and (5) p27 (T198). **(G)** Western blot analysis confirmed that phosphorylated PKB/Akt proteins is downregulated in HK2-silencing HNSCC cells. p38, P38 mitogen-activated protein kinase; LDHA, lactate dehydrogenase A. **(H)** Graphic summary of HK2-mediated regulations in HNSCC cells. Data are presented as mean ± SEM (*N* > 3). ***P < 0.001.

HK2-mediated metabolic and molecular regulators for HNSCC malignancy were next examined. Metabolic reprogramming was recently proposed, suggesting that cancer cells could adapt to unfavorable metabolic stresses (73). Numbers of investigations have been reported showing metabolic shift upon HK2 changes in non-HNSCC cells (32, 40, 50, 54); in agreement with these findings, Seahorse analysis showed that HK2 loss led to increased basal and maximal mitochondrial respiration (Figures 5A,B), implying HK2-silencing HNSCC cells were metabolically programmed, being more dependent on mitochondrial metabolism. This statement could be further supported by the results showing a greater extracellular acidification rate (ECAR) upon addition of ATPase inhibitor oligomycin (Figure 5C) and increased PDH activity (Figure 5D) in HK2-silencing HNSCC cells. Unexpectedly, HK2 deficiency–mediated decreased cell growth did not result from reduction of cellular ATP level (Figure 5E), suggesting that bioenergetics could be dispensable for metabolism-mediated cell growth. Using a high-throughput human protein array, it was discovered that Akt (S473), TP53 (S15, S46, and S392), and p27 (T198) proteins were downregulated in HK2-silencing OECM-1 cells (Figure 5F). Western blot analysis further confirmed that Akt activity, but not another common oncogenic signaling factor Erk1/2, was deceased in HK2-deficient OECM-1 and HSC3 cells (Figure 5G).

## Discussion

In the present study, it was shown that HK2 loss downregulated cell growth, EMT-mediated cell movement, and stemness/differentiation-related treatment sensitivity; in addition, altered bioenergetics and modulation of oncogenic signaling molecules including Akt and phosphorylated TP53 could underlie HK2-mediated regulations in HNSCC cells (Figure 5H). Overall findings concluded that targeting HK2 could be an ideal therapeutic strategy in treating HNSCCs. One interesting discovery from the current study is that in HNSCC cells HK2-mediated malignant suppression is likely through glycolytic manipulation based on the following data: (i) HK2 is highly expressed in HNSCC cells implying that HNSCCs are “glycolytic” tumor; (ii) HK2 inhibition triggers metabolic shift away from glycolysis toward mitochondrial metabolism and (iii) it is found that mitochondria are swelling and lack cristae in HNSCC cells compared with human oral fibroblasts (Supplementary Figure 5). This finding agreed with previous investigations showing that HK2-mediated metabolic modulation is a key machinery to control cancerous characteristics (41, 54, 74, 75), even though it is required to further define the potential impact of mitochondrial HK2 (49, 51) in controlling tumorigenesis in HNSCC cells in the current experimental setting.

Another interesting finding is the potential involvement of phosphorylated TP53 and p27 proteins in HK2-mediated regulations for HNSCC oncogenicity. Based on protein array analysis, HK2 loss resulted in decreased phosphorylated TP53 at transactivation domain (S15 and S46) and C-terminal domain (S392) in OECM-1 cells. While previous sequencing analysis for TP53 gene in OECM-1 cells showed that OECM-1 cells carry mutant TP53 (P139R/V239L) (62), phosphorylation on S15, S46, and S392 of TP53 could be oncogenic in OECM-1 cells. This statement could be supported by previous studies showing that S15 phosphorylation of TP53 contributed mutant TP53 stability, thereby prolonging cell viability in ovarian cancer cells (76), and S392 phosphorylation in mutant TP53 could lead to tumor progression in esophageal squamous cell carcinoma (77). While it was reported that T198 phosphorylation of cell cycle regulator p27 promotes cyclin D1–CDK4–p27 complex assembly (78), as well as increases p27-RhoA–mediated cell motility (79), HK2 silencing–mediated T198 p27 loss could be a potential underlying cue in controlling HNSCC cell growth and migration. Further elucidation for roles of different TP53 phosphorylation sites in both wild-type and mutant HNSCC cells in regulating tumorigenicity is expected.

With extensive studies, including the present investigation, showing that HK2 is a master promoting factor in different stages of carcinogenesis (Supplementary Table 4), much efforts were put in exploring HK-mediated tumor inhibitors, despite that no potent drugs targeting HK2 are yet available in clinic (80–84). Taking the most promising HK2-targeting drug, 3-bromopyruvate (3-BP), as an example, although single treatment of 3-BP, at the appropriate dose and formulation, could effectively destroy glycolytic tumors and seems to be non-toxic to all sorts of vertebrates, certain failure cases were still reported in clinical test (83). It is noteworthy that 3-BP administration also resulted in great pain and inflammation in an HNSCC patient enrolled in a 3-BP trial at the Dayspring Cancer Clinic (http://www.dayspringcancerclinic.com). In addition to a demand of a larger clinical cohort, to achieve better outcomes by using 3-BP or other HK2 inhibitors, as stated in Supplementary Table 4, to cure cancer in clinic, a very recent article suggested that simultaneous or sequential treatment of 3-BP and other conventional therapeutic agents needs to be tested for optimal tumor-killing ability, and/or localized delivery of 3-BP directly into tumor tissues should be considered without affecting normal cells (85). Based on current findings, a more systemic study to test synergic antiproliferative effects of different HK2 inhibitors, not only for 3-BP but also for other known small molecules (listed in Supplementary Table 4), and their underlying molecular mechanisms in combination of treatments of chemotherapeutic drugs, radiotherapy, and targeted therapy (such as EGFR antagonist cetuximab), needs to be conducted, both *in vitro* and *in vivo*. In addition to BP-3, it is hoped that other inexpensive and safe anticancer HK2 inhibitors could also be discovered, successfully pass clinical trials, and soon be available in global market.

## Data Availability Statement

The datasets generated for this study are available on request to the corresponding author.

## Ethics Statement

The studies involving human participants were reviewed and approved by Institutional Review Boards (IRB) of MacKay Memorial Hospital and National Taiwan University Hospital, Taiwan. The patients/participants provided their written informed consent to participate in this study. This animal study was reviewed and approved by Institutional Animal Care and Use Committee (IACUC), National Yang-Ming University.

## Author Contributions

W-CL devised and coordinated the study. C-HH, Y-TH, T-YC, L-HC, and C-YC performed experiments. C-HH conducted animal experiments. C-HH and Y-TH performed statistical analysis. C-JL, H-MC, C-LH, J-FL, and K-WC provided samples and critical discussion. W-CL wrote the manuscript. All authors read and approved the final manuscript.

### Conflict of Interest

The authors declare that the research was conducted in the absence of any commercial or financial relationships that could be construed as a potential conflict of interest.
