# Corrigendum: Regulatory Role of Hexokinase 2 in Modulating Head and Neck Tumorigenesis

**DOI:** 10.3389/fonc.2020.00410

**Published:** 2020-03-31

**Authors:** Wan-Chun Li, Chien-Hsiang Huang, Yi-Ta Hsieh, Tsai-Ying Chen, Li-Hao Cheng, Chang-Yi Chen, Chung-Ji Liu, Hsin-Ming Chen, Chien-Ling Huang, Jeng-Fan Lo, Kuo-Wei Chang

**Affiliations:** ^1^Institute of Oral Biology, School of Dentistry, National Yang-Ming University, Taipei, Taiwan; ^2^Department of Dentistry, School of Dentistry, National Yang-Ming University, Taipei, Taiwan; ^3^Cancer Progression Research Center, National Yang-Ming University, Taipei, Taiwan; ^4^Department of Oral and Maxillofacial Surgery, MacKay Memorial Hospital, Taipei, Taiwan; ^5^Department of Medical Research, MacKay Memorial Hospital, Taipei, Taiwan; ^6^School of Dentistry and Department of Dentistry, National Taiwan University Medical College and National Taiwan University Hospital, Taipei, Taiwan; ^7^Department of Health Technology and Informatics (HTI), The Hong Kong Polytechnic University (PolyU), Kowloon, Hong Kong; ^8^Department of Stomatology, Taipei Veterans General Hospital, Taipei, Taiwan

**Keywords:** head and neck cancer, hexokinase 2, cellular malignancy, metabolic shift, therapeutic efficacy

An author name was incorrectly spelled as “**Jen-Fang Lo**.” The correct spelling is “**Jeng-Fan Lo**.” The authors apologize for this error and state that this does not change the scientific conclusions of the article in any way. The original article has been updated.

