# Supplementary material for: Regulatory Role of Hexokinase 2 in Modulating Head and Neck Tumorigenesis
Source: Front Oncol. 2020 Mar 3;10:176. doi: 10.3389/fonc.2020.00176 (PMC7063098; doi:10.3389/fonc.2020.00176)
Supplement: Supplementary file 1 [file Presentation_1.PPTX]

## Slide 1
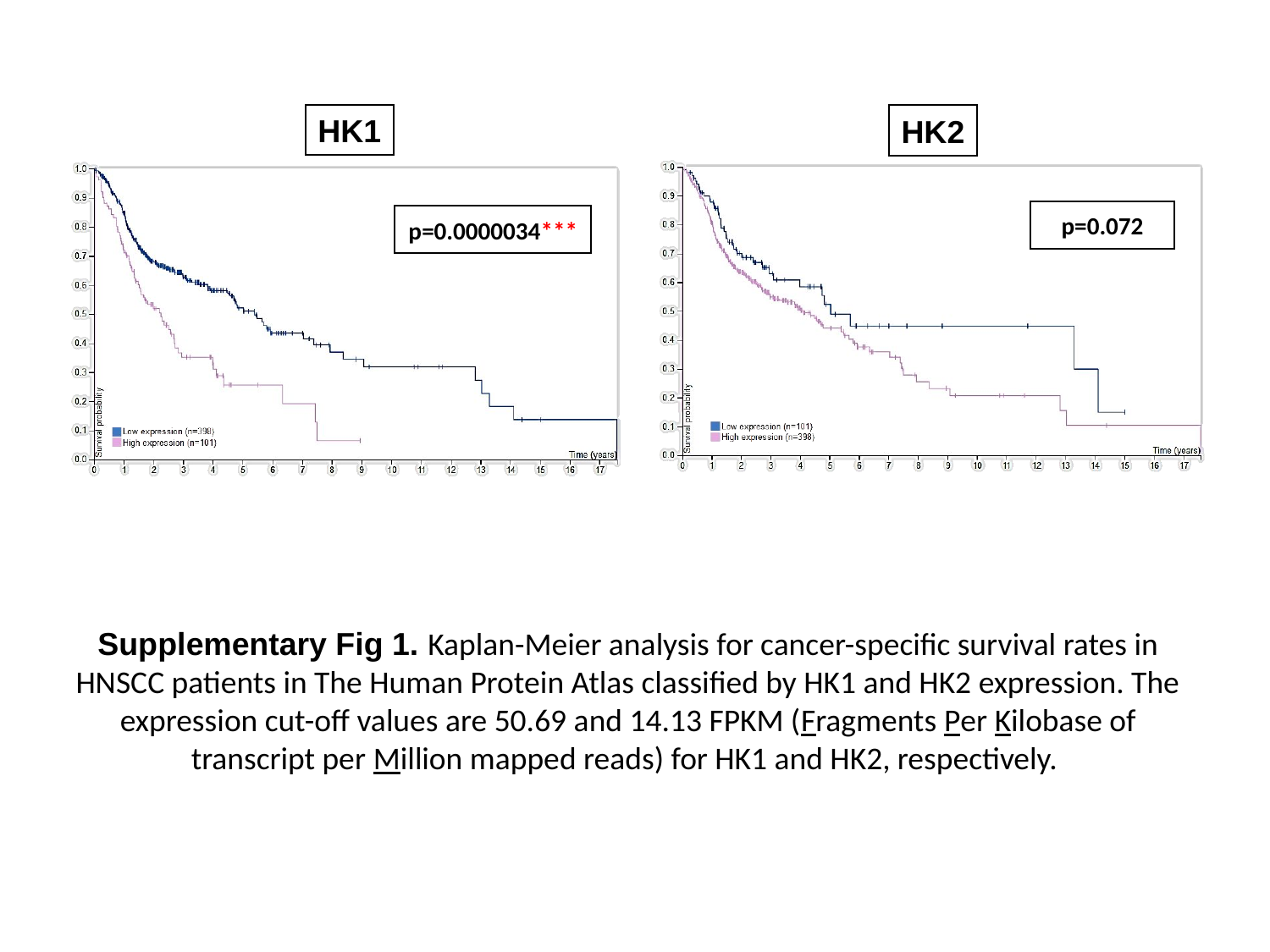

HK1
HK2
p=0.072
p=0.0000034***
Supplementary Fig 1. Kaplan-Meier analysis for cancer-specific survival rates in HNSCC patients in The Human Protein Atlas classified by HK1 and HK2 expression. The expression cut-off values are 50.69 and 14.13 FPKM (Fragments Per Kilobase of transcript per Million mapped reads) for HK1 and HK2, respectively.

## Slide 2
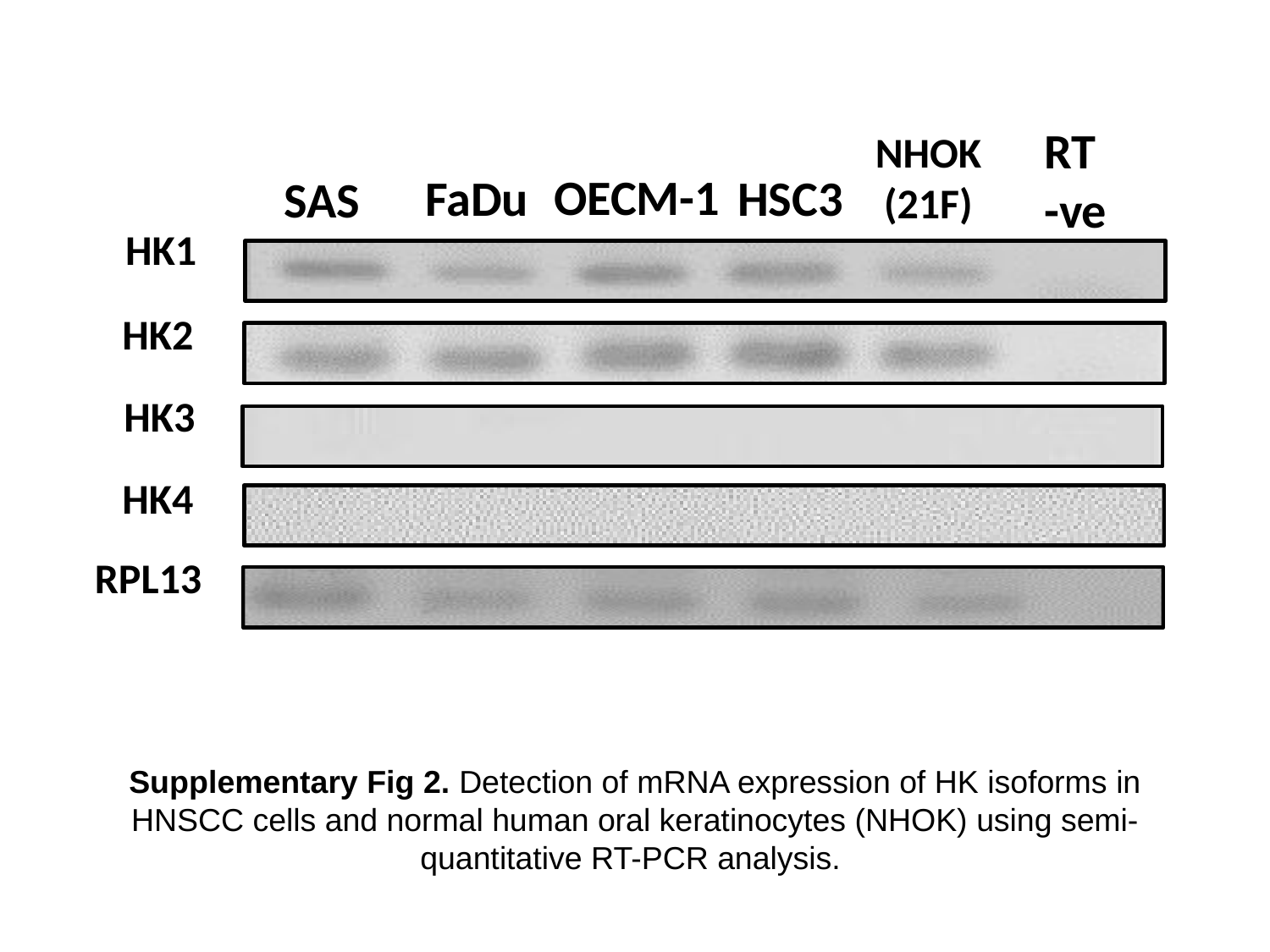

RT
-ve
NHOK
(21F)
OECM-1
HSC3
FaDu
SAS
HK1
HK2
HK3
HK4
RPL13
Supplementary Fig 2. Detection of mRNA expression of HK isoforms in HNSCC cells and normal human oral keratinocytes (NHOK) using semi-quantitative RT-PCR analysis.

## Slide 3
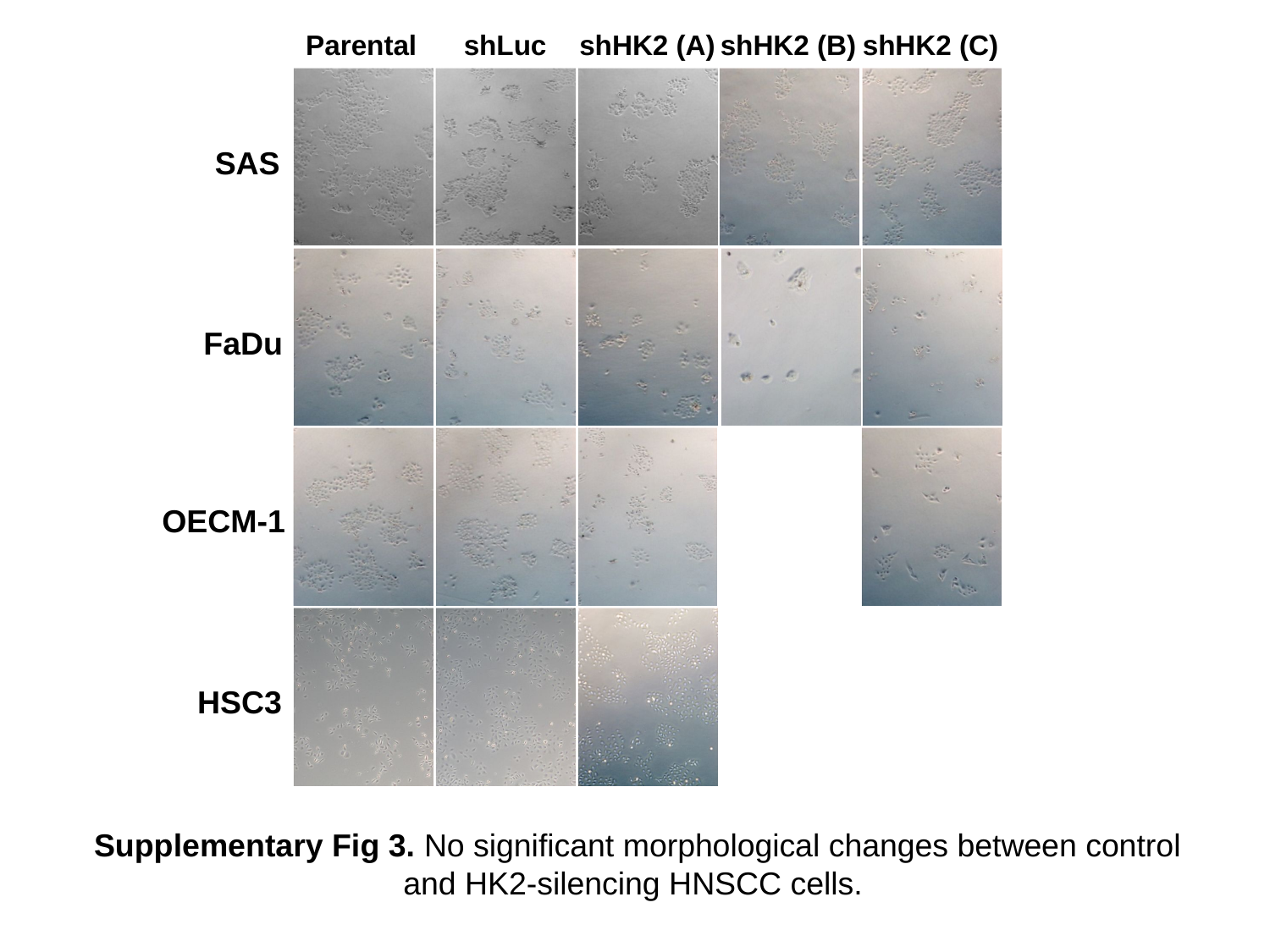

Parental
shLuc
shHK2 (A)
shHK2 (B)
shHK2 (C)
SAS
FaDu
OECM-1
HSC3
Supplementary Fig 3. No significant morphological changes between control and HK2-silencing HNSCC cells.

## Slide 4
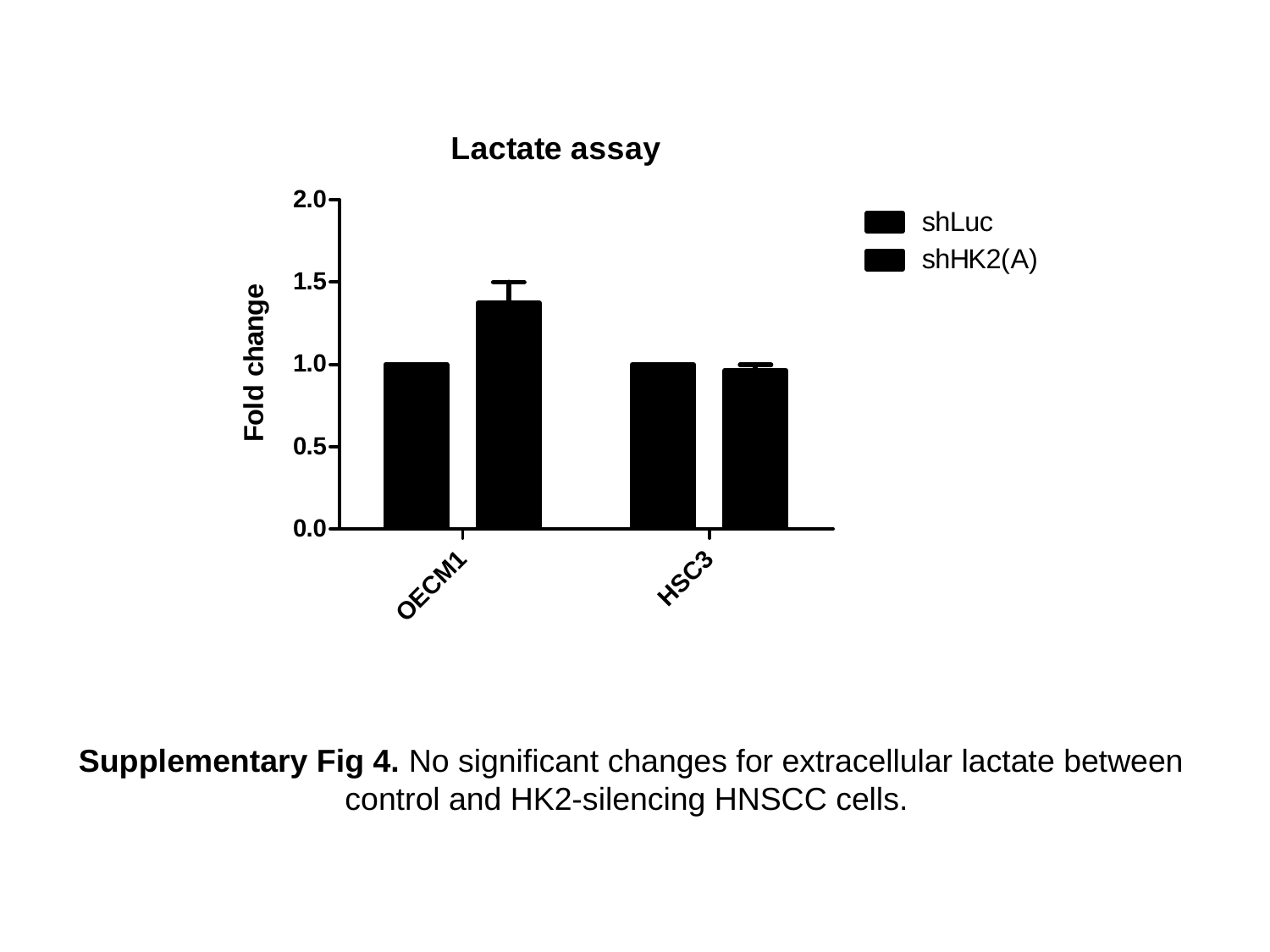

Supplementary Fig 4. No significant changes for extracellular lactate between control and HK2-silencing HNSCC cells.

## Slide 5
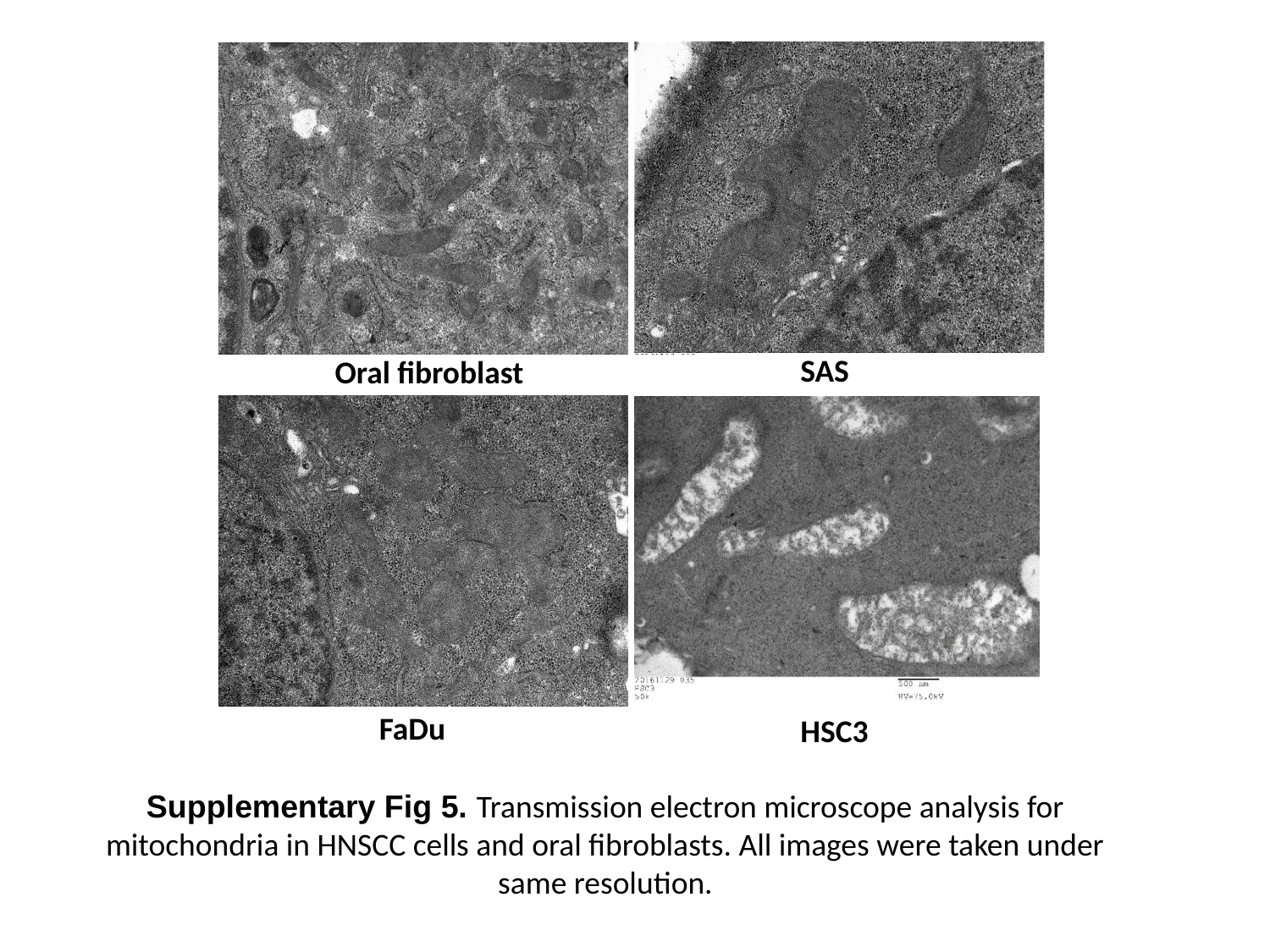

SAS
Oral fibroblast
FaDu
HSC3
Supplementary Fig 5. Transmission electron microscope analysis for mitochondria in HNSCC cells and oral fibroblasts. All images were taken under same resolution.

## Slide 6
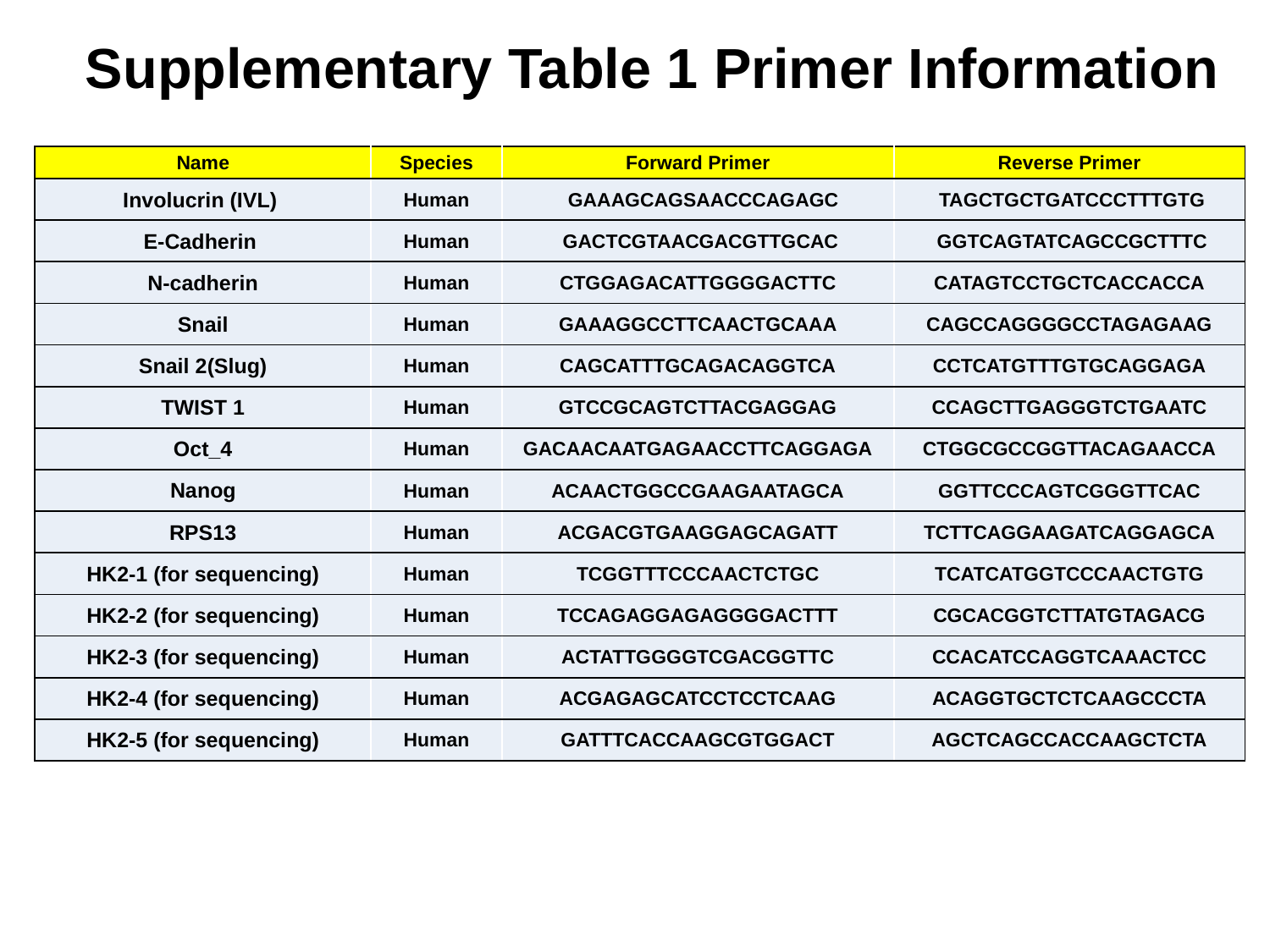

Supplementary Table 1 Primer Information
| Name | Species | Forward Primer | Reverse Primer |
| --- | --- | --- | --- |
| Involucrin (IVL) | Human | GAAAGCAGSAACCCAGAGC | TAGCTGCTGATCCCTTTGTG |
| E-Cadherin | Human | GACTCGTAACGACGTTGCAC | GGTCAGTATCAGCCGCTTTC |
| N-cadherin | Human | CTGGAGACATTGGGGACTTC | CATAGTCCTGCTCACCACCA |
| Snail | Human | GAAAGGCCTTCAACTGCAAA | CAGCCAGGGGCCTAGAGAAG |
| Snail 2(Slug) | Human | CAGCATTTGCAGACAGGTCA | CCTCATGTTTGTGCAGGAGA |
| TWIST 1 | Human | GTCCGCAGTCTTACGAGGAG | CCAGCTTGAGGGTCTGAATC |
| Oct\_4 | Human | GACAACAATGAGAACCTTCAGGAGA | CTGGCGCCGGTTACAGAACCA |
| Nanog | Human | ACAACTGGCCGAAGAATAGCA | GGTTCCCAGTCGGGTTCAC |
| RPS13 | Human | ACGACGTGAAGGAGCAGATT | TCTTCAGGAAGATCAGGAGCA |
| HK2-1 (for sequencing) | Human | TCGGTTTCCCAACTCTGC | TCATCATGGTCCCAACTGTG |
| HK2-2 (for sequencing) | Human | TCCAGAGGAGAGGGGACTTT | CGCACGGTCTTATGTAGACG |
| HK2-3 (for sequencing) | Human | ACTATTGGGGTCGACGGTTC | CCACATCCAGGTCAAACTCC |
| HK2-4 (for sequencing) | Human | ACGAGAGCATCCTCCTCAAG | ACAGGTGCTCTCAAGCCCTA |
| HK2-5 (for sequencing) | Human | GATTTCACCAAGCGTGGACT | AGCTCAGCCACCAAGCTCTA |

## Slide 7
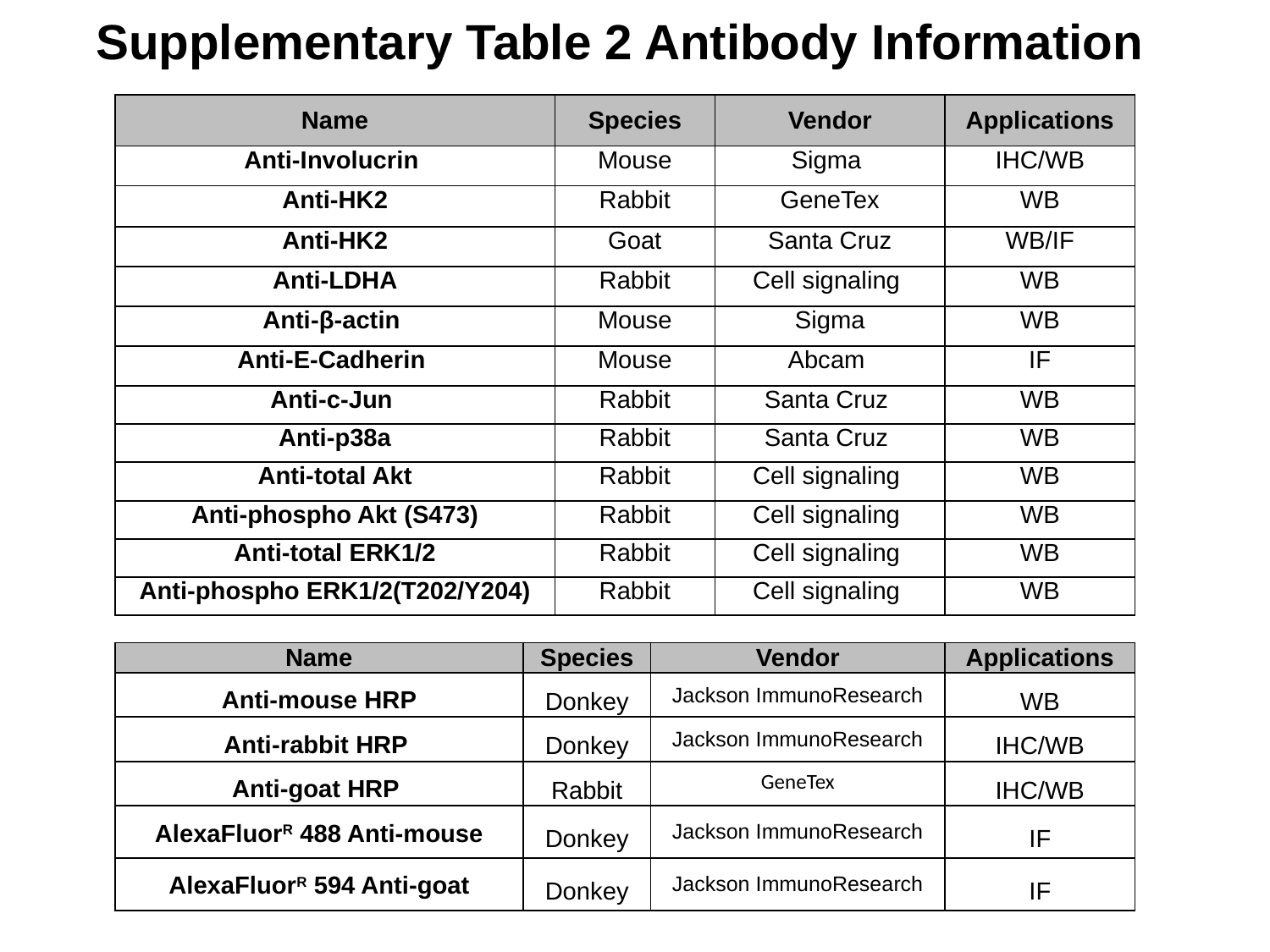

Supplementary Table 2 Antibody Information
| Name | Species | Vendor | Applications |
| --- | --- | --- | --- |
| Anti-Involucrin | Mouse | Sigma | IHC/WB |
| Anti-HK2 | Rabbit | GeneTex | WB |
| Anti-HK2 | Goat | Santa Cruz | WB/IF |
| Anti-LDHA | Rabbit | Cell signaling | WB |
| Anti-β-actin | Mouse | Sigma | WB |
| Anti-E-Cadherin | Mouse | Abcam | IF |
| Anti-c-Jun | Rabbit | Santa Cruz | WB |
| Anti-p38a | Rabbit | Santa Cruz | WB |
| Anti-total Akt | Rabbit | Cell signaling | WB |
| Anti-phospho Akt (S473) | Rabbit | Cell signaling | WB |
| Anti-total ERK1/2 | Rabbit | Cell signaling | WB |
| Anti-phospho ERK1/2(T202/Y204) | Rabbit | Cell signaling | WB |
| Name | Species | Vendor | Applications |
| --- | --- | --- | --- |
| Anti-mouse HRP | Donkey | Jackson ImmunoResearch | WB |
| Anti-rabbit HRP | Donkey | Jackson ImmunoResearch | IHC/WB |
| Anti-goat HRP | Rabbit | GeneTex | IHC/WB |
| AlexaFluorR 488 Anti-mouse | Donkey | Jackson ImmunoResearch | IF |
| AlexaFluorR 594 Anti-goat | Donkey | Jackson ImmunoResearch | IF |

## Slide 8
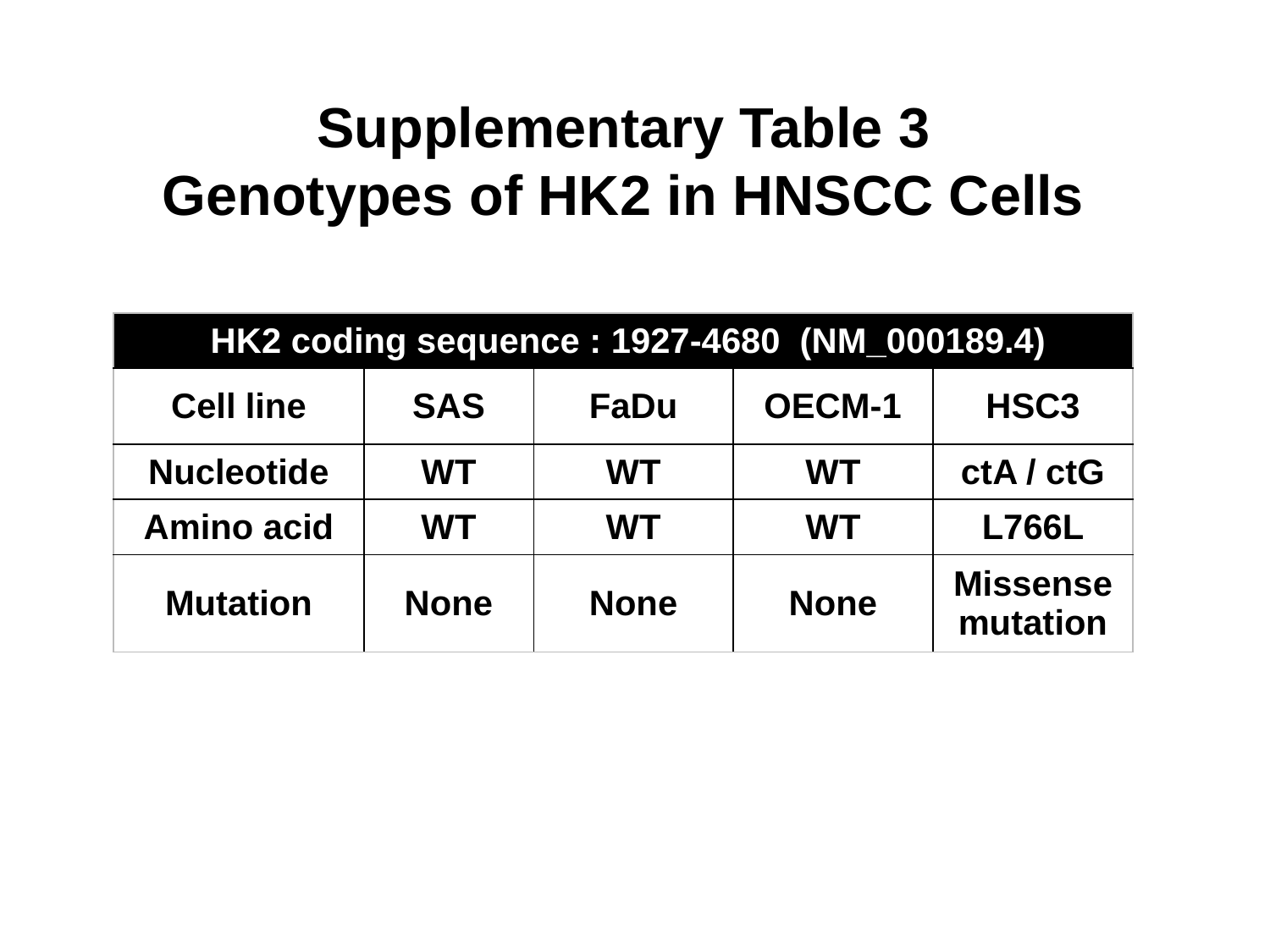

Supplementary Table 3
Genotypes of HK2 in HNSCC Cells
| HK2 coding sequence : 1927-4680 (NM\_000189.4) | | | | |
| --- | --- | --- | --- | --- |
| Cell line | SAS | FaDu | OECM-1 | HSC3 |
| Nucleotide | WT | WT | WT | ctA / ctG |
| Amino acid | WT | WT | WT | L766L |
| Mutation | None | None | None | Missense mutation |
